# The Evolution of Communication and Education Strategies of Canadian Transplant Programs During the Pandemic

**DOI:** 10.1177/15269248231189865

**Published:** 2023-07-18

**Authors:** Shaifali Sandal, Elie Fadel, Emilie Trinh, Michael Gagnon, Andrea Herrera-Gayol, Marcelo Cantarovich

**Affiliations:** 1 Division of Nephrology, Department of Medicine, McGill University Health Centre, Montreal, Quebec, Canada; 2 Research Institute of the McGill University Health Centre, Montreal, Quebec, Canada; 3 Division of Experimental Medicine, Department of Medicine, McGill University Health Centre, Montreal, Quebec, Canada; 4 Scientific Consultant, Montreal, Quebec, Canada

**Keywords:** COVID-19 pandemic, transplantation, patient care, videoconference, interprofessional communication

Communication breakdowns are key barriers in access to transplantation and effective education of patients and treating teams are critical to optimal patient care.^1,2^ Despite this, limited work has been done to identify optimal education and communication strategies.^3^ In 2019, we pursued a quality improvement initiative to improve communication and education between Canadian transplant programs and community specialists, primary care physicians (PCPs) and patients. A short survey was administered electronically to representatives of all adult and pediatric transplant programs (kidney: 27, heart: 16, lung: 7, liver: 10) in Canada from April to May 2019. Then the COVID-19 pandemic presented an unprecedented urgency to adopt virtual approaches. We utilized this serendipitous opportunity to examine how the previously identified strategies evolved during the pandemic. Following approval from the Research Ethics Board of the McGill University Health Centre, we administered the same survey from July to October 2021 with two additional questions.

The response rates for the first and second survey were 98.3% and 86.7%, respectively. If participants were unavailable or declined to take part, an alternative person was contacted. A descriptive analysis of the survey responses data was conducted, and we used chi-square to compare proportions that chose videoconference as a strategy pre-pandemic and during the pandemic.

**Figure 1.**
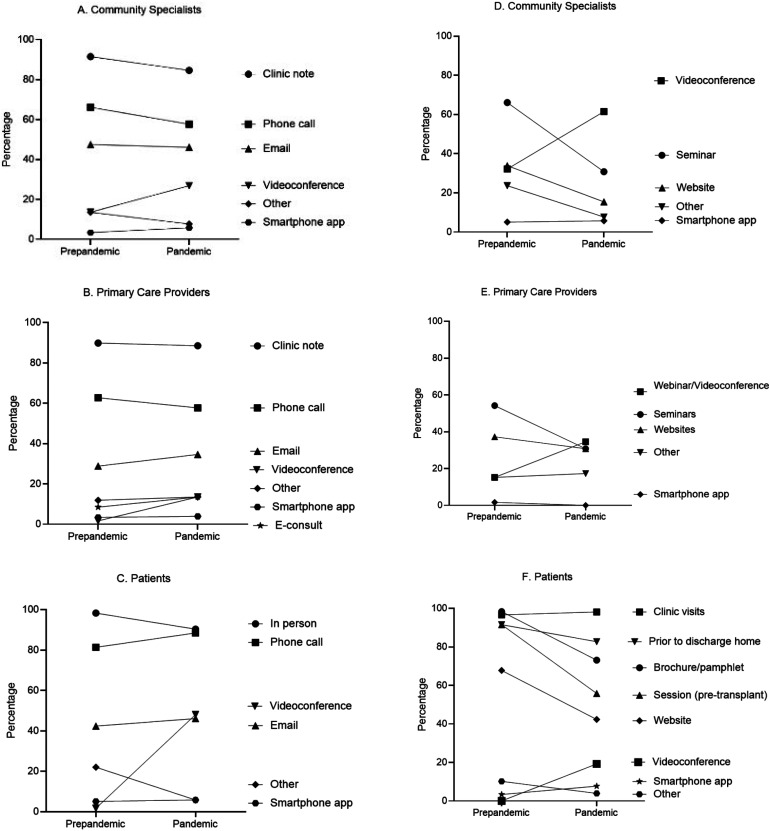
The evolution of communication (A, B, C) and education (D, E, F) strategies of transplant programs prepandemic and during the pandemic with community specialists, primary care physicians, and patients, respectively.

Over half of the participants (53.9%) agreed/strongly agreed that better communication strategies were developed during the pandemic while 19.2% disagreed/strongly disagreed and 26.9% were neutral. The proportion of programs that used videoconference with community specialists doubled (prepandemic: 13.6% vs pandemic: 26.9% *P *= .08), and there was a significant increase in its use when communicating with PCPs (prepandemic: 1.7% vs pandemic: 13.5%, *P *= .02) and patients (prepandemic: 1.7% vs pandemic: 48.1%, *P *< .001). Despite this, traditional methods, such as clinic notes and phone calls, continued to be the top choices (**Figure 1A-C)**.

Only a third of the participants (32.7%) agreed/strongly agreed that better education strategies were developed during the pandemic while 25.0% disagreed/strongly disagreed and 42.3% were neutral. Videoconference was among the top 3 strategies in use both prepandemic and during the pandemic for community specialists and PCPs and a significant increase was noted; prepandemic: 32.2% versus pandemic: 61.5% (*P *= .002) and prepandemic: 15.2% versus pandemic: 34.6% (*P *= .02), respectively. For patient education, the proportion increased from 0% prepandemic to 19.2% during the pandemic (*P *< .001). Multiple approaches were employed for patient education in the prepandemic times, but there appeared to be a decline during the pandemic **(Figure 1D-F)**.

The most important finding of our study was that despite the unprecedented need to adopt virtual approaches during the COVID-19 pandemic, most transplant programs continue to use traditional methods to communicate and educate community specialists, PCPs, and patients. While there was a significant increase in the use of videoconference during the pandemic, there exist barriers to universal adoption. We also report that fewer opportunities for in-person visits may have led to a decline in patient education. Thus, only a third agreed that better education strategies were developed. Our work offers a reflection on communication and education strategies in Canadian transplant programs and that capacity building and addressing barriers to the uptake of telehealth are needed.
